# Elevated Smooth Pursuit Gain in Collegiate Athletes with Sport-related Concussion Immediately Following Injury

**DOI:** 10.18502/jovr.v19i2.12348

**Published:** 2024-06-21

**Authors:** Madison R. Taylor, Marian Berryhill, Dennis Mathew, Nicholas G. Murray

**Affiliations:** ^1^School of Public Health, University of Nevada, Reno, NV, USA; ^2^Department of Psychology, University of Nevada, Reno, NV, USA; ^3^Department of Biology, University of Nevada, Reno, NV, USA

**Keywords:** Concussion, mTBI, Oculomotor Control, Visual Acuity

## Abstract

**Purpose:**

Although there is evidence that sport-related concussion (SRC) affects oculomotor function and perceptual ability, experiments are often poorly controlled and are not replicable. This study aims to test the hypothesis that there are decreased values when assessing oculomotor impairment indicating poorer performance in SRC patients.

**Methods:**

Fifteen DI athletes presenting with SRC (7 females, 8 males) and 15 student volunteers (CON) (12 females, 3 males) completed a dynamic visual acuity (DVA) task that involved answering the direction of a moving stimulus (Landolt C) while wearing a head-mounted binocular eye tracker. There were 120 trials total with 60 trials presenting at 30º per second and 60 presenting at 90º per second. Various eye movement measurements, including horizontal smooth pursuit eye movements (SPEM) gain and saccadic peak velocity, were analyzed between groups using univariate ANOVAs. Saccade count in SPEM trials, accuracy, and vision were analyzed using Kruskal–Wallis tests.

**Results:**

There was no statistical difference in saccadic peak velocity: SRC = 414.7 ± 42º/s, CON = 406.6 ± 40.6º/s. A significant difference was found between SRC patients and healthy controls in horizontal SPEM gain (SRC = 0.9 ± 0.04, CON = 0.86 ± 0.03, F(1,28) = 7.243, *P* = 0.012) indicating that patients demonstrated compensatory eye movements when tracking the target. There were significantly more saccades in all SPEM trials (*P* = 0.001).

**Conclusion:**

SRCoculomotor deficits manifest as elevated horizontal SPEM gain when assessed within 48 hours of injury and compared to healthy controls within the same age range**. **SRC demonstrates altered oculomotor ability. While accurate in tracking a stimulus, SRC patients may conduct less controlled eye movements.

##  INTRODUCTION

Sport-related concussion (SRC) is a significant public health concern, accounting for approximately 21% of all mild traumatic brain injuries (i.e., concussion) with > 50% going unreported.^[1]^ Despite monitoring and assessment of SRC at universities and colleges, the impact of SRC on visual acuity (VA) and oculomotor ability, both of which are crucial aspects of an athlete's routine, may be overlooked. Mild traumatic brain injury (mTBI) is often interchangeably used with concussion to refer to a blow to the head, typically resulting in metabolic impairments of the brain. Symptoms can range from headache, fatigue, emotional disruption, and decreased cognitive function and are a direct result of the metabolic imbalance following an injury.^[2,3]^

The visual system spans 50% of the human brain, making it a vital component in the diagnosis of SRC. Moreover, those who present with oculomotor symptoms, for example, vestibular (dizziness, nausea, balance issues) or atypical vision (blurred vision, diplopia, and headaches) are at greater risk of protracted recovery from SRC.^[4,5]^ If brain regions vital to vision are impaired, clinical tests can detect these dysfunctions within the midbrain, potentially presenting as an inability to perceive movement.

A common clinical assessment that measures vestibular/ocular function already exists, yet it relies heavily on a self-report system. The Vestibular/Oculomotor Screening (VOMS) uses a series of subtests that tracks symptom provocation, or the presence of four common SRC symptoms (i.e., headache, dizziness, nausea, fogginess).^[6]^ The VOMS is useful in diagnosing and monitoring SRC, but is ultimately a subjective measure at risk of administrator error in addition to an athlete's underreporting of their symptoms.^[7]^ Video-oculography is a powerful laboratory tool that can measure particular eye movements such as fixations, smooth pursuit eye movements (SPEMs), and saccades. If the experiment is properly controlled, the eye movement data can specify abnormal functions in neuronal pathways.^[8]^ Video-oculography shows that eye movement deficits occur in up to 90% of all SRC cases.^[9,10]^ However, this method is expensive and inaccessible to clinicians. Therefore, research must continue to bridge the gap between clinical visual tests (i.e., the VOMS) and eye tracking following SRC to understand the neuropathology behind SRC.

Two well-defined and studied eye movements that are likely affected following SRC are SPEMs, slower eye movements; and saccades, faster eye movements.^[11,12,13]^ Abnormal eye movements can lead to downstream impairments, such as poor motor responses when performing typical athletic movements.

In several studies, researchers observed group differences in SPEM metrics between SRC and healthy controls.^[14,15]^ A previous study observed SRC requiring more catch-up saccades during SPEM trials; however, they did not disclose the number of saccades performed.^[12]^ In SRC, saccadic eye movements are generally greater in number, are poorly controlled, and have greater velocity.^[16,17,18,19,20,21,22]^ Generally, the most deficits in saccades following SRC are noted during self-paced and memory-guided saccades.^[20]^

However, these studies lack well-controlled and replicable stimuli, appropriate reporting of psychometrics of their results, injury-related features (i.e., time since injury and clinical symptoms/vision tests), and behavioral data. This study overcomes these issues by using a lower-order task (dynamic visual acuity [DVA]) that is easily replicable and incorporates various other metrics that will aid in determining possible deficits present in SRC.

The purpose of this study was to investigate group differences in collegiate SRC and a healthy population. We expect these differences to manifest as reduced DVA, indicating poorer performance in tracking and perceiving a moving stimulus among SRC subjects. We expect the SRC will demonstrate greater saccadic velocities, more catch-up saccades in SPEM trials, and decreased SPEM gain.

**Figure 1:**
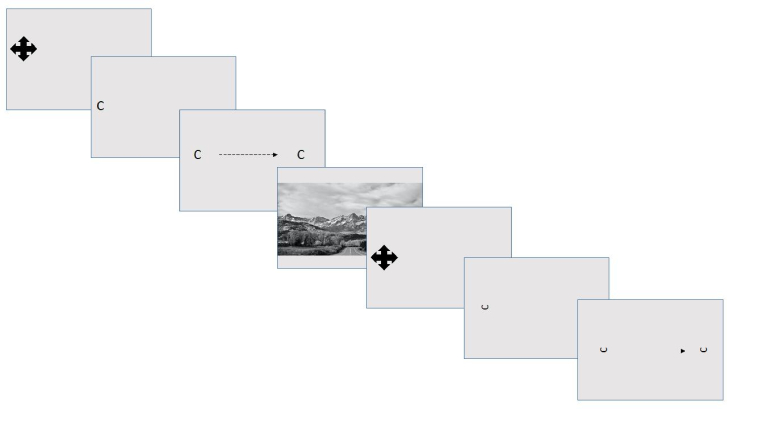
Depiction of DVA task. During the task, the c moves in a horizontal position (left to right) starting with the presentation of a fixation cross. A retinal flush image is presented between trials to reduce retinal smearing.

**Figure 2:**
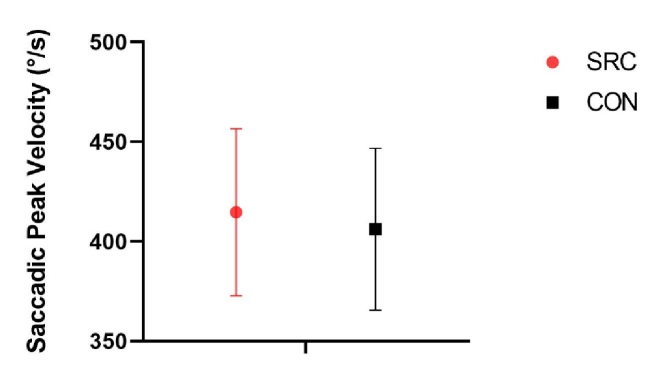
Saccadic Peak Velocity (SPV) of the sport-related concussion (SRC) and control (CON) participants. No significant difference is noted between the groups.

**Figure 3:**
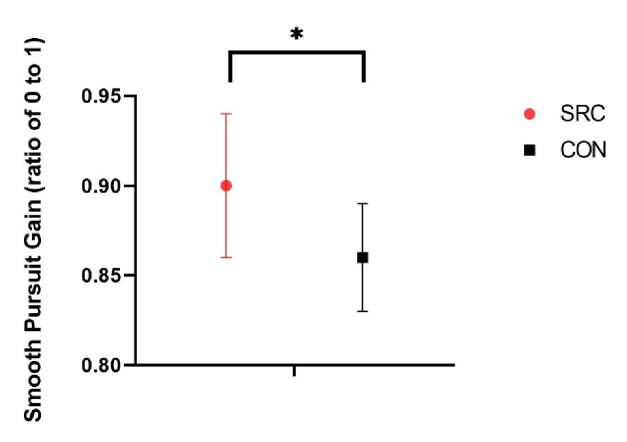
Smooth Pursuit Eye Movement (SPEM) gain of sport-related concussion (SRC) and control (CON) participants. No significant increase is noted between the groups.

**Figure 4:**
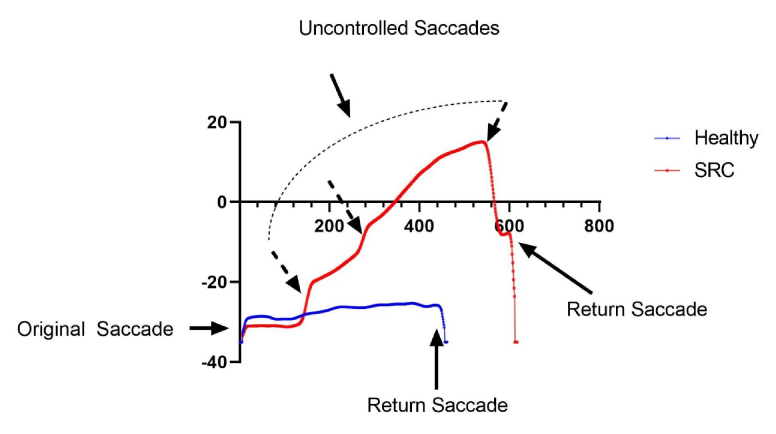
Mapped raw eye trace data from one SRC and one CON participant. Note the uncontrolled intrusive saccades in a SPEM trial for the SRC participant.

**Table 1:** Average LogMAR values for the static and each dynamic stimuli for each group with Snellen scores reported for ease of interpretation


	**SRC (** ***n*** ** = 10)**	**CON (** ***n*** ** = 14)**
Static LogMAR (Snellen)	–0.08 (20/16)	–0.13 (20/16)
SPEM LogMAR (Snellen)	0.12 (20/25)	0.11 (20/25)
Saccade LogMAR (Snellen)	0.32 (20/40)	0.26 (20/40)
	
	
CON, control; LogMAR, logarithm of the minimum angle of resolution; SRC, sport-related concussion patient		

**Table 2:** Mean, standard deviation, and intraclass correlation coefficient for saccadic peak velocity, horizontal SPEM gain, SPEM LogMAR, and saccadic LogMAR using the Eyelink II at two different time points.


	**Saccadic peak velocity (degrees/sec)**	**Horizontal SPEM gain**	**SPEM LogMAR**	**Saccadic LogMAR**
Day 1	441.2 ± 25	0.89 ± 0.07	0.09 ± 0.09	0.28 ± 0.06
Day 2	435.6 ± 29	0.87 ± 0.05	0.13 ± 0.13	0.31 ± 0.12
ICC	0.94 (0.42 to 0.996)	0.90 (–0.629 to 0.993)	0.74 (–3.011 to 0.98)	0.05 (–13.738 to 0.938)
	
	
ICC, intraclass correlation; LogMAR, logarithm minimum angle of resolution SPEM, smooth pursuit eye movements				

**Table 3:** Kruskal–Wallis H values and *P*-values for total number of saccades, average number of saccades per SPEM trials, and accuracy for all trials, SPEM trials, and saccades trials


	**Total saccades in SPEM trials**	**Average number of saccades per SPEM trial **	**Total trials accuracy**	**SPEM trial accuracy**	**Saccade trial accuracy**
SRC Average and SD values	160.07 ± 33.13	2.67 ± 0.62	0.64 ± 0.19	0.69 ± 0.18	0.67 ± 0.17
CON Average and SD values	106 ± 21.93	1.8 ± 0.41	0.73 ± 0.08	0.74 ± 0.03	0.74 ± 0.04
Cohen's d	1.925	1.656	–0.613	–0.4	–0.556
P-value	< 0.001	< 0.001	0.086	0.474	0.475
	
	
CON, controls; SD, standard deviation; SPEM, smooth pursuit eye movements; SRC, sport-related concussion					

##  METHODS

Fifteen National Collegiate Athletic Association (NCAA) Division I student-athletes diagnosed with SRC by the head team physician using consensus statement recommendations were included (7 females, 8 males; age = 20 years). Athletes were evaluated within 1–48 hours post-injury. Fifteen student non-athlete volunteers were recruited as healthy controls (CON) (12 females, 3 males; age = 21 years) at pre-season physicals. Participants with the following conditions were excluded from the study: abnormal vision, prior diagnosed neurological injury within six months, attention-deficit hyperactivity disorder, any existing or persisting symptoms related to a COVID-19 infection, learning disabilities, currently pain-free beyond normal exercise-induced soreness and any lower extremity injury that could impair the ability to stand upright as determined by self-report. All protocols were approved by the University of Nevada, Reno Institutional Review Board in the USA, and all participants signed an informed consent document prior to participating (IRB Number: 1757959-8).

Participants completed an oral initial symptom checklist, tandem gait walking, and the VOMS. Only the VOMS will be reported here as the other variables will be reported in a larger study. VOMS consists of several subtests that evaluate SPEM, horizontal and vertical saccades, near-point convergence, horizontal and vertical vestibular ocular reflex, and visual motion sensitivity. After each subtest, SRC symptoms are ranked on a scale of 0 to 10, described as 0 being typical and 10 being extreme. A change score was calculated by summing the total amount of symptom increase from baseline scores. A change score of ≥ 2 is considered a positive test result for vision and/or vestibular impairment following SRC.^[23]^

The VOMS was administered in VR with an HTC Vive Pro Eye Head Mounted Display (HMD) with a diagonal focus of vision (FOV) of 110º, refresh rate of 90 Hz, a combined resolution of 2880 × 1600 pixels, six degrees of freedom (DoF) for position and orientation tracking, and adjustable interpupillary (IPD) and focal distances. The participant's nose length was measured and recorded to account for HMD. The headset was powered by an Acer Predator gaming laptop with a 7th Generation Intel Core i7 Quad-Core processor with 16 GB of memory and NVIDIA GeForce GTX 1070 graphics card running Windows 10. The HTC VIVE 2.0 Hand Controller received input from participants to initiate and stop the stimulus movement when convergence occurred during near-point convergence (NPC). This method has been validated in a healthy population.^[24]^

SVA was assessed using the Freiburg Visual Acuity Test (FrACT).^[25]^ The participant sat 154 cm away from the screen, fixating on the letter E presented in one of four orientations (up, down, left, or right). Participants pressed the arrow key on a standard keyboard corresponding to the perceived orientation. For the dynamic task, the participants sat 154 cm away from a 26º display screen (Pixio, 165 Hz, 2560 × 1440 resolution, Torrance, CA, USA) wearing a head-mounted eye tracker (Eyelink II, 500 Hz, SR Research Inc., ON, Canada). The eye-tracking system was calibrated using a 13-point calibration matrix with the head stabilized in a chin rest. A black Landolt-C traveled from left to right on a gray background in one of four orientations with equal probability of each (up, down left, or right) [Figure1]. The participant was instructed to track the C and report the direction of the opening as quickly as possible. The size of the C began at a LogMAR of 1 (Snellen 20/200) and could get as small as a LogMAR of –0.2 (Snellen 20/12.5) depending on performance in a one-up-two-down staircase method. There were 120 trials, with 60 presenting at a velocity of 30º/s to elicit SPEMs and 90º/s to elicit saccades.

We conducted an additional reliability experiment to investigate DVA and the Eyelink II. Four healthy non-athletic participants (average age = 23 years) with normal or corrected to normal vision were first screened using the FrACT SVA and then performed the DVA experiment. The participants repeated the DVA task within 24 hours. We used repeated measures ANOVAs to find within-groups differences in the variables collected by the Eyelink II, including saccadic velocities, horizontal SPEM gain, and LogMAR values for both SPEM and saccades. Intraclass coefficients (ICC) were calculated using the data.

SVA was calculated by taking responses and calculating at what optotype the patient was 60% accurate.^[25]^ This output calculated a LogMAR value, which was converted into a Snellen score for ease of interpretation. Both are reported in this study.

A custom MATLAB 2021b code (The Mathworks, Natick, MA) analyzed the raw eye data and transformed it into azimuth and elevation, then interpolated and smoothed it. In the 60 SPEM trials, a temporal window of 10–30 ms was used to remove amplitudes below 0.66º, eliminating fixations and saccades.^[26,27]^ A velocity threshold filtered velocities between 10º/s and 30º/s.^[16]^ SPEM gain was calculated as the ratio of eye velocity to stimulus velocity such that a gain value of 1 reflected perfect foveation of the moving target.

A 5-point linear interpolation and a 13-point sliding boxcar filter were used to analyze saccades. Data were filtered using a dispersion threshold between 4º/s and 30º/s.^[16]^ The dispersion data were divided by the temporal length of the trial and further filtered using a velocity threshold between 75º/s and 500º/s to determine velocity.^[16]^

The SPEM trials of each participant were manually plotted to demonstrate the velocity of the eye throughout the trial. They were manually combed for catch-up saccades by counting the number of times the velocity jumped higher than 35º/s for an average of 30 ms with a significant amplitude change ( ≥ 4). These counts were totaled for the 60 trials and averaged per trial for each group.

One-way ANOVAs separately analyzed peak saccadic velocity, horizontal smooth pursuit gain, and NPC. A multivariate analysis analyzed SVA LogMAR, SPEM LogMAR, and saccadic LogMAR. All analyses compared between-group (SRC, CON) performances. Total number of saccades, average number of saccades per SPEM trials, and accuracy for all trials, SPEM trials, and saccades were independently analyzed using Kruskal–Wallis tests.

##  RESULTS

Eight SRC and six CON reported symptom provocation ( ≥ 2 provoked symptoms) on the VOMS. NPC was not significantly different between SRC (2.9 cm) and CON (3.5 cm).

Average SPV values for SRC showed no significant group difference (*P* = 0.6, Cohen's d = 0.2) than CON (SRC = 414.7 ± 42º/s, CON = 406.6 ± 40.6º/s). Horizontal SPEM gain values were significantly higher (F( ${\;}_{1,28}$ ) = 7.243, *P* = 0.012, Cohen's d = 1.1) in the SRC group (0.9 ± 0.04) compared to CON (0.86 ± 0.03) [Figure 2].

Of all psychometric data, only 10 SRC and 14 CON produced enough accurate responses to produce a curve. Among the participants that did generate a curve, there was no significant difference among LogMAR values in SVA (SRC = –0.08 ± 0.2, CON = –0.13 ± 0.04, *P* =0.4), SPEMs (SRC = 0.12 ± 0.2, CON = 0.11 ± 0.1, *P* = 0.82), or saccades (SRC = 0.25 ± 0.2, CON = 0.25 ± 0.1, *P* = 0.37) [Figures2 & 3].

There were significantly higher saccades present in all SRC SPEM trials than in CON (SRC = 160.07 ± 33.13, CON = 106 ± 21.93, *P* < 0.001, Cohen's d = 1.925). There were also significantly higher saccades in SPEM trials when averaged per trial for SRC (SRC = 2.67 ± 0.62, CON = 1.8 ± 0.41, *P* < 0.001, Cohen's d = 1.656) [Table 3]. We graphed each eye trace and provided a visual representation of SRC and CON [Figure4].

From the data collected in the reliability data subset, there was no significant difference between two separate time points, verifying the reliability of the Eyelink II hardware and software for use in the general population through analysis of ICCs [Table *2*]. The saccadic LogMAR demonstrated the lowest ICC and all values should be interpreted with caution using that metric.

##  DISCUSSION

The purpose of this study was to investigate the differences in eye movement impairments immediately following SRC compared to healthy controls. The data suggest a clear discrimination in SPEM gain and a slight non-significant difference in saccadic peak velocity. The most important finding of this study is that following SRC, SPEM gain increased which could be partially driven by the number of saccades initiated during the task [Figure 4]. This suggests poor motion perception, as the participants are requiring additional saccades to increase the foveation time to discern the stimuli. While the overall vision for the SPEM trials was not different between groups, it is clear that following SRC, more saccades are needed during a horizontal tracking task in order to properly foveate. With excessive catch-up saccades, SRC may be unable to successfully track the stimuli during the task due to a reduction in foveation [Table3].

However, SRC and CON were similar in accuracy for SPEMs, saccades, and all trials. We analyzed vision, seen in Table 1, using psychometric curves, finding the size optotype that the participant correctly guessed 62.5% of the time.^[28]^ One of the fifteen control participants did not produce a psychometric curve for smooth pursuits, while 5 of the 15 SRC patients did not. The participants who did not produce a curve did not have enough correct answers per optotype to fit a proper psychometric curve, so these data were excluded from this analysis. While the SRC group was able to more accurately track the stimulus, data suggests that an overall reduction in the ability to see the stimuli occurred. This is evidenced by the increased number of saccades in the SPEM trials and that 5 of the 15 SRC did not produce enough correct responses to generate a psychometric curve.

Another possible explanation for our data is the presence of glutamate immediately after SRC. Research in mice suggests there is an increased secretion of glutamate within the first 48 hours following injury.^[29]^ Because these patients are evaluated within a timeline of 48 hours, these results could be explained by the heightened activity in the brain due to the excess glutamate in the brain. ${\;}^{\text{[34]}}$ This should be explored further to determine the role glutamate may play within the first 48 hours of SRC.

Of the data reported, some variables did not differ significantly, indicating they are not appropriate measures in diagnosing concussion. Findings supported prior literature that saccadic velocity does not differ, indicating SRC does not alter the ability to perform a saccade.^[20,30]^ Moreover, NPC has been a common method of diagnosing SRC, but these findings do not support that claim.^[6]^

This study was not without limitations. The fixed horizontal stimulus may have caused the athletes to fixate on one area of the screen, knowing the exact location the stimulus would originate from and travel to. As we are reliant on naturally occurring head injuries, we were limited by sample size and sport-specific SRC diagnoses, which could explain our insignificant results. Future research should utilize a larger and more diverse sample of athletes for more accurate results that are generalizable to the population. In addition, saccadic intrusions were removed from the SPEM data and future investigations should evaluate the total number and length of these. In addition, the controls in this study heavily featured females. While sex doesn't appear to influence eye movements, further research should match controls based on age and sex to the experimental participants. Finally, this study utilized intermediate VA, instead of far VA, as it was more appropriate for the presented stimulus. Future research should incorporate more clinically accessible measures of VA.

In conclusion, our findings indicate that immediately after injury, SRC can cause elevated SPEM gain values when compared to healthy controls which may be caused by hyperactivity or heightened levels of glutamate. This is imperative to understand when treating and diagnosing SRC in a collegiate setting, potentially able to generalize to other sport populations.

##  Financial Support and Sponsorship

The authors would like to thank the UNR Neuroscience COBRE (P20GM103650) for funding this research.

##  Conflicts of Interest

None.
